# Developmental differences in the impact of perceptual salience on short-term memory performance and meta-memory skills

**DOI:** 10.1038/s41598-022-11624-8

**Published:** 2022-05-17

**Authors:** Tiziana Pedale, Serena Mastroberardino, Michele Capurso, Simone Macrì, Valerio Santangelo

**Affiliations:** 1 Functional Neuroimaging Laboratory, IRCCS Santa Lucia Foundation, Rome, Italy; 2 Department of Psychology, School of Medicine and Psychology, Sapienza University of Rome, Rome, Italy; 3 Department of Philosophy, Social Sciences and Education, University of Perugia, Piazza G. Ermini 1, 06123 Perugia, Italy; 4 Centre for Behavioural Sciences and Mental Health, Istituto Superiore di Sanità, Rome, Italy

**Keywords:** Short-term memory, Spatial memory, Attention

## Abstract

In everyday life, individuals are surrounded by many stimuli that compete to access attention and memory. Evidence shows that perceptually salient stimuli have more chances to capture attention resources, thus to be encoded into short-term memory (STM). However, the impact of perceptual salience on STM at different developmental stages is entirely unexplored. Here we assessed STM performance and meta-memory skills of 6, 10, and 18 years-old participants (total N = 169) using a delayed match-to-sample task. On each trial, participants freely explored a complex (cartoon-like) scene for 4 s. After a retention interval of 4 s, they discriminated the same/different position of a target-object extracted from the area of maximal or minimal salience of the initially-explored scene. Then, they provided a confidence judgment of their STM performance, as an index of meta-memory skills. When taking into account ‘confident’ responses, we found increased STM performance following targets at maximal versus minimal salience only in adult participants. Similarly, only adults showed enhanced meta-memory capabilities following maximal versus minimal salience targets. These findings documented a late development in the impact of perceptual salience on STM performance and in the improvement of metacognitive capabilities to properly judge the content of one’s own memory representation.

## Introduction

Everyday life is rich of stimuli that may need to be processed by our cognitive system because of their potential relevancy for goal-directed behaviour. Attention plays a critical role in stimulus selection and in the prioritization of stimulus elaboration^1–3^. Visual (or perceptual) “salience” has been shown to be a good proxy to estimate attention selection (e.g.^4–9^), especially when these processes are investigated in complex and real-life situations rich of stimuli competing for processing resources (e.g., real-world visual scenes or videos^10^). Perceptual salience can be defined as the distinct subjective perceptual quality which makes some items more attentional-capturing than others. Consistently, “saliency maps” (i.e., two-dimensional topographical maps that encode stimulus conspicuity, or salience, at every location of the visual scene^11,12^) were shown to successfully predict patterns of eye movements during free viewing of complex scenes (i.e., using both pictures^4,6,7^ and videos^5,8,9^).

Importantly, not only does perceptual salience predict overt exploration (i.e., eye movements) of visual scenes, but also encoding prioritization (for a review, see^13^). Specifically, perceptual salience has been shown to affect post-perceptual processing, such as short-term memory (STM), whereby perceptually salient objects have more chances to be encoded and then successfully retrieved than objects with lower salience^14–20^. For instance, Santangelo & Macaluso^19^ used a delayed match-to-sample task in which participants were presented, at encoding, with everyday life scenes. At retrieval, participants had to discriminate the location (same vs. different) of a target object that was extracted from the initial scene. Crucially, the target object could be selected from either the area of maximal or minimal perceptual salience, as defined by the “saliency maps” computed for each of the initially-explored scenes^11,12^. The results showed that individual performance at retrieval was remarkably more accurate when, at encoding, the to-be-judged target was located at the point of maximal salience in the scene, indicating encoding prioritization for salient information.

While the above-mentioned observations refer to findings obtained in adulthood, the impact of perceptual salience on encoding prioritization and STM performance at different developmental stages was—at the very best of our knowledge—entirely neglected by the previous literature. Although a number of studies demonstrated a gradual developmental improvement of STM capacity, especially from 3 to 10 years old^21–28^, they did not take into account the specific link between perceptual salience and STM. Developmental studies investigated the role of perceptual salience only at the attentional level, e.g. during scene‐viewing, and provided conflicting results. Some of these studies reported that salience is a better predictor for adults’ than for infants’ eye movements^29^, showing that, in general, the predictive value of perceptual salience increases as a function of age (e.g.^30–35^). However, other studies reported the opposite pattern of results. For instance, Frank and colleagues^36^ showed that saliency predicts eye movements better than faces in younger infants (3‐month‐olds), whereas in older infants (6‐ and 9‐month‐olds), faces became a better predictor than salience (for similar results, see also^37,38^). Finally, some other recent evidence suggests instead that there is no difference between the impact of perceptual salience on eye movements in infancy and adulthood^39^. While these mixed findings can originate from specific differences at the methodological level, whether perceptual salience can affect also post-perceptual processes (such as encoding prioritization and STM performance) to a different extent at various developmental stages is currently unexplored.

The development of the interplay between attention selection and STM performance was recently explored by Shimi and Scerif^27^ adopting an ontogenetic perspective (see^40–43^ for studies investigating the interplay between attention and memory on adult subjects). Shimi and Scerif administered 6–7 years-old (y.o.) children and young adults a delayed match-to-sample task in which they manipulated both memory load and attention control (i.e., endogenous attention using a spatial pre-cue). They found that both processes (attention and memory load) synergically contributed to the efficiency of STM performance in adults versus children. In the current study, we investigated the interplay between attention selection and STM performance from a new perspective, using complex and naturalistic displays, wherein attentional selection was unconstrained and modelled in terms of the perceptual salience of the to-be-remembered object. Moreover, we investigated whether perceptual salience differentially affects memory-related metacognitive skills, i.e., the capability to correctly estimate the content of own memory, at various developmental stages. Several factors can affect memory-related metacognitive judgments (for a review, see^44^). One of these is related to “encoding fluency”, which predicts that items that are easier to be encoded tend to promote more confident predictions regarding memory performance, even in the absence of actual better memory performance^45,46^. This effect has been mostly investigated by using lists of words, but recently also a “perceptual” fluency effect has been demonstrated. Rhodes and Castel^47^ presented participants with words with different font sizes (large- vs. small-fonts). When participants were asked to predict their subsequent memory performance, they predicted better performance for larger (vs. smaller) font words, that is, for those items that were easier to be perceptually processed. This effect suggested that the mere perceptual appearance of the stimuli affected memory confidence, even when this was irrelevant to the memory task. Here, we further investigated the effect of perceptual fluency on metacognitive judgments using visual scenes to test whether higher perceptual salient objects—that are easier to be processed/encoded—elicited higher confidence in metamemory judgments. Moreover, this effect could dramatically vary according to the participants’ age. It is widely accepted that the improvement of metacognitive skills is a fundamental component of cognitive development (for reviews, see^48,49^), and researchers have started to specifically investigate meta-memory skills, showing a direct link between them and memory performance. In fact, meta-memory skills were shown to be associated with better learning^50,51^ and better estimation of own memory, whereby they may promote the implementation of better mnemonic strategies^52^. In line with this view, Jones and colleagues^53^ recently reported that a working memory training accompanied by a metacognitive strategy training is far more efficient in increasing academic outcomes in 9–14 y.o. children than the working memory training alone. Moreover, a growing body of literature on the development of meta-memory abilities across age suggests a higher tendency in overestimating own memory abilities in children compared to young adults^54,55^. In the current study, we aim to glean a more complete picture of the development of meta-memory skills abilities at different ages, by investigating whether the higher versus lower perceptual salience of the to-be-remembered target would differentially affect the correct estimation of one own’s memory performance as a function of developmental stage.

We compared STM performance of young and older children of 6 and 10 y.o., respectively, as previous evidence indicated dramatic development changes of both STM capacity (e.g.^21^) and meta-memory skills (e.g.^54^) across these ages. Children’s performance was compared to a group of young adults (18 y.o.) using a delayed match-to-sample task. On each trial, participants freely explored a complex (cartoon-like) scene for 4 s. After a retention interval of 4 s, they discriminated the same versus different position of a target object extracted from the initial scene, which could have been located within the area of maximal or minimal salience of the scene. Then, participants were asked to provide a confidence judgment in which they evaluated their own STM performance, as an index of meta-memory skills. On the basis of the available literature, we hypothesised a progressive increase in STM performance and meta-memory abilities from childhood to early adulthood. Given the mixed findings related to the effect of perceptual salience on attention selection across age, we aim to further explore whether salience affects STM performance and meta-memory skills to a similar or a different extent from childhood to adulthood.

Finally, we also assessed whether STM performance was affected by the overall complexity of the scene. A previous study by Cavallina and colleagues^56^ revealed that scene complexity, indexed by the “number of salient locations” in the scene, affects the deployment of spatial attention during a visual search task. This was especially true for younger children (6 y.o.), who showed decreased performance as a function of increased scene complexity. This effect was interpreted as due to a “dispersion” of attentional resources when coping with highly complex stimuli. Given this intriguing result, we decided to take into account the possible impact of scene complexity also in the current STM task, since it could have an influence on the global processing of the initially-explored scenes. Given the negative impact of complexity on the deployment of attentional resources^56^, we expected decreased STM performance and meta-memory confidence when scene complexity increases. Additionally, our experimental design allowed addressing whether these effects varied with target saliency and participants’ age.

## Methods and materials

### Participants

A total of 170 healthy participants volunteered for and took part in the study. One young child failed to understand the task, leaving a final sample of 169 participants, including 63 young children attending the first grade (27 males; mean age: 6.2 y.o., range: 6–7 y.o.), 67 older children attending the fifth grade (33 males; mean age: 10 y.o., range: 9–11 y.o.), and a control group of 39 young adults (15 males; mean age: 18.5 y.o., range: 17–20 y.o.). The children were recruited across three Comprehensive Schools (“Giovan Battista Valente”, Rome, Italy; “Enzo Giuliani” and “Umberto Fifi”, Bastia Umbra, Perugia, Italy), while the adults were recruited from the University of Perugia. The minimum sample size for this study was estimated a priori with G*Power 3.1.9.2. For the ANOVAs (repeated measures, within-between interaction) we took into account a conservative estimate of the effect size of 0.2 (based on previous studies investigating the interplay between attention and STM at developmental level; see^21,27^), a power of 95%, a significance level of 0.05, 3 groups, 2 measurements (i.e., the two salience conditions), a correlation among repeated measures of 0.5, and nonsphericity correction of 1. This indicated a minimum sample size of 34 participants per group. The exclusion criteria for children included a diagnosis of neurodevelopmental disorders, as reported by parents or teachers. All participants had a normal or corrected-to-normal vision and were naïve to the main purpose of the study, which was approved by the independent Ethics Committee of the IRCCS Santa Lucia Foundation (CE/PROG.665) and conducted in adherence to the tenets of the Declaration of Helsinki. All adult participants provided informed consent. Parental consent was obtained for each child participant.

### Stimuli and task

**Figure 1:**
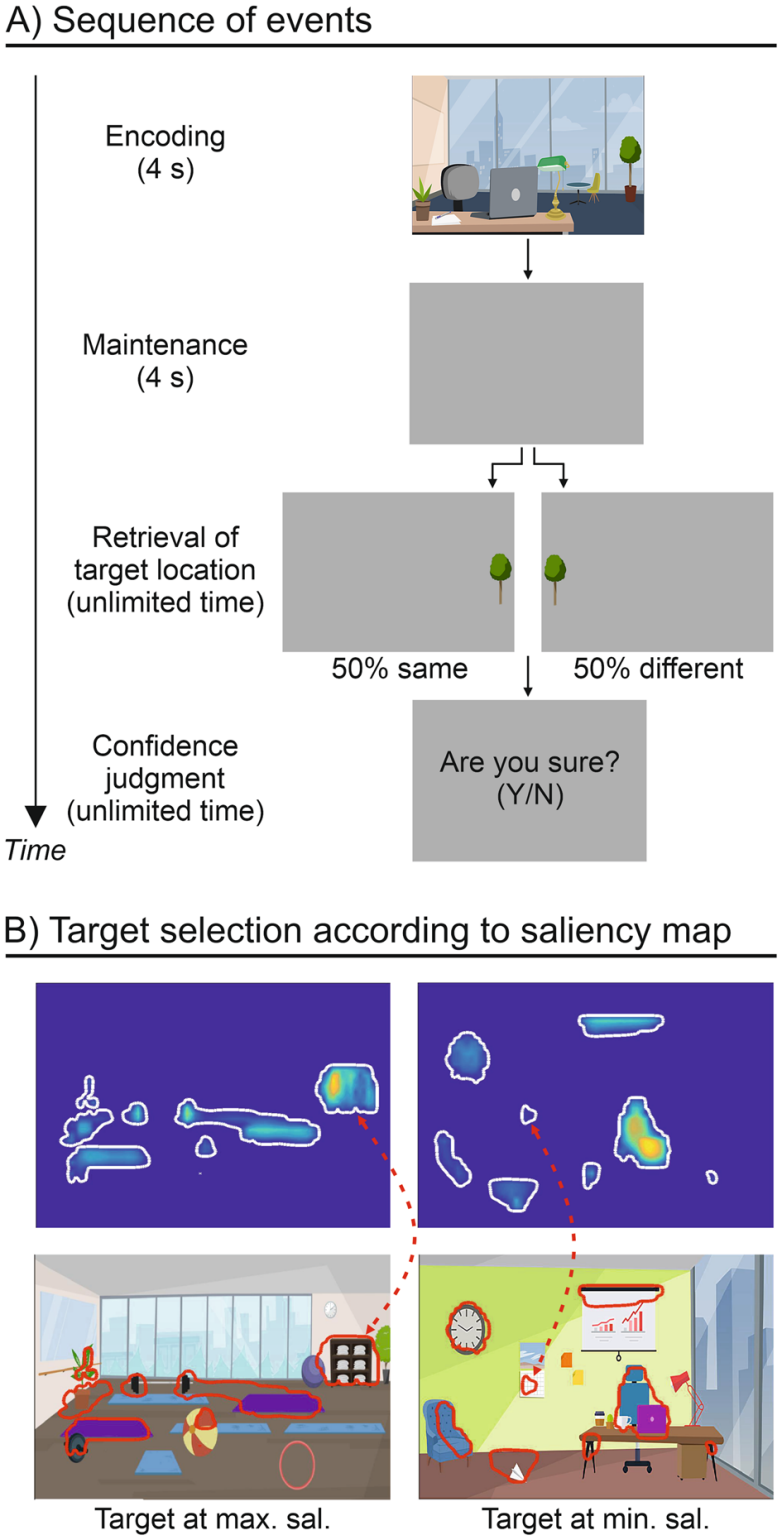
Experimental protocol. (**A**) Schematic diagram showing the sequence of events during one trial. The trial began with a cartoon scene presented for 4 s (encoding). A grey display was then shown for 4 s (maintenance). At retrieval, a single target object was presented either in the same or different location compared to the object’s location in the original picture. With unlimited time, participants had to judge the same versus different location of the target and then provide a confidence judgment requiring a yes/no response, as an index of meta-memory skills. (**B**) Examples of two cartoon scenes showing the selection of the target object related to either the point of maximal (left) or minimal salience (right) of the scene.

Participants sat in a quiet room in front of a laptop computer. The laptop display was placed approximately 50 cm from the viewer (picture size = 29 × 22° of visual angle). Each trial of the task consisted of an encoding phase (4 s), a maintenance phase (4 s delay), a retrieval phase (discrimination of target-object location; unlimited time), and, finally, a memory confidence judgment (unlimited time, see Fig. 1A). At the encoding phase, subjects were presented with cartoon scenes created using GoAnimate (GoAnimate^©^ 2016; https://goanimate.com/) at a resolution of 1280 × 720 pixels. The use of cartoon scenes was chosen to increase the attractiveness of the task for the younger groups of participants (for a similar approach, see^57^). The cartoon scenes depicted scenes of everyday life, involving either internal (e.g., a kitchen, a bathroom, etc.) or external (e.g., a garden, a street, etc.) backgrounds. Each scene included several objects, but no living elements, such as people or animals, to avoid the inclusion of socially relevant stimuli that could spontaneously capture attention resources irrespective of perceptual salience^58^. At encoding, participants were required to memorize as many details as possible of the visual scene for later retrieval. For each scene, a target object was designed for being presented at retrieval, but the identity of the target object was unknown until the retrieval phase. Following the 4 s delay (blank screen), participants were administered the retrieval phase (discrimination of target-object location). This consisted of the presentation of a designed target object cut from the original picture and pasted on a grey background, using CorelDraw Graphics Suite v. 12 (Copyright^©^ 2016, Corel Corporation; https://www.coreldraw.com/). Two versions of the retrieval display were created for each scene: one in which the target object was presented at the same location as in the original image (“same” condition), and one in which the target object was presented at the mirror location in the opposite hemifield (“different” condition; note that only the object location was flipped, but the object itself remained unchanged, as well as the position in terms of the vertical axis; see Fig. 1A). The participants’ task consisted of reporting whether the target object presented at the retrieval phase was in the “same” versus “different” location with respect to the original scene explored at encoding by pressing on the computer keyboard “J” for “same” and “N” for “different”. The participants had unlimited time to answer. The decision to allow unlimited time to provide the answer was chosen to make the task easier for the younger participants, thus avoiding potential errors due to time pressure. In half of the trials, the target object was presented at the same location, while in the other half of the trials the target object was presented at the opposite location. After the retrieval phase, participants were required to provide a memory-related metacognitive judgment concerning their response to the target discrimination task. For this, a display with the question “Are you sure? (y/n)” was presented, and participants pressed “J” for “yes” and “N” for “no” response, again with unlimited time to answer. Between each trial, participants were presented with an inter-trial interval (ITI) of 1 s consisting of a grey background. Before the experimental session, participants practiced with a short training session consisting of 6 trials, involving cartoon scenes not used in the main experiment.

The experiment included 48 trials, each making use of a different cartoon scene. Each scene was analysed through the Saliency Toolbox 2.2 (http://www.saliencytoolbox.net/). This created a saliency map for each scene using local discontinuities in line orientation, intensity contrast, and color opponency^11^. The saliency maps allowed us to select as targets for the retrieval phase either the object located within the area of minimal or maximal perceptual salience in the visual scene (see Fig. 1B). In half of the scenes, the designed target was the object located within the area of maximal perceptual salience (“Max” condition, 24 trials); while in the other half of the scenes, the designed target was the object located within the area of minimal perceptual salience (“Min” condition, 24 trials). Moreover, for each scene, we computed the “number of salient locations” (NSL), i.e., an index of the complexity of the scene (cf.^56^), which was extracted from the above-described saliency maps, and consisted of the number of saliency peaks included within each scene (this index included also the saliency peak that corresponded to the target object). The NSL ranged from a minimum of 6 to a maximum of 13 peaks. For each condition (Max and Min), there were 3 scenes for each possible NSL (i.e., 3 scenes with 6 salient locations, 3 scenes with 7 salient locations, 3 scenes with 8 salient locations, and so on).

To rule out the possibility that saliency-related effects were confounded by other perceptual factors, we computed two additional indices for each scene, namely “target size” and “horizontal target eccentricity” (cf.^20,59,60^). The target size can be defined as the spatial area occupied by the target (i.e., the proportion of pixels within the target-object with respect to the overall number of pixels of the picture). The horizontal target eccentricity can be instead defined as the distance (converted in degrees of visual angle) between the vertical meridian of the scene and the horizontal coordinate of the center of mass of the target. The latter was computed by averaging the horizontal and vertical coordinates of each pixel belonging to the target object (see, e.g.^18^). To avoid that the target size and the horizontal target eccentricity could affect the saliency related results, we checked and confirmed that these perceptual factors did not significantly differ between the scenes belonging to the two saliency conditions (i.e., the 24 scenes in which the target-object was located within the area of maximal saliency, Max condition, versus the 24 scenes in which the target-object was located within the area of minimal perceptual saliency, Min condition): target size: *t*(46) = 0.29, *p* = .77; horizontal target eccentricity: *t*(46) = − 0.19, *p* = .84.

### Data analysis

We collected responses corresponding both to the retrieval memory task (“correct” or “incorrect” discrimination of the target location) and to the following confidence judgment (“Yes, I’m sure” or “No, I am not sure” responses). First, we calculated the percentage of correct responses irrespective of the confidence judgment (‘Correct’). Then, to confirm the results obtained with the ‘Correct’ data, we computed an ‘Accuracy sensitivity’ measure of memory performance, namely, the d-prime, according to the formula: d-prime = z (hits rate) − z (false alarms rate)^61^. “Hits” were defined as targets that appeared at retrieval in the same position as at encoding and that were correctly identified (“same” response); while “false alarms” were defined as targets located in a different position at retrieval that were incorrectly recognized as matching the original scene (“same” response). The d-prime score is important since the participants were always required to provide a response to the memory retrieval task (cf. the unlimited response time) to move to the confident judgment and then to the next trial. It might be therefore possible that a certain amount of target discrimination responses was—by chance—correct even when participants had no target memory at all. A d-prime score of 0.0 indicates that the detection of the target position was at a chance level, while a positive d-prime indicates better-than-chance memory performance.

Then, we computed the percentage of responses that were both accurate and sure, i.e., correct discriminations of the target location followed by a “Yes, I’m sure” response (‘Correct & Sure’; see, for an analogous approach^18,19,62^). With this index, we computed the percentage of responses that were not only merely accurate but also relied on a memory trace that was strong enough to make a “Sure” judgment. In the training session, we emphasized to the participants to press “Yes, I’m sure” at the confident judgment phase, if—and only if—they were confident about their previous response to the target spatial discrimination task, and to press “No, I’m not sure” when they responded at chance. For these reasons, the combined computation of both correct and confident responses can be considered as the pivotal conjunction measure between the two cognitive mechanisms (i.e., memory and metacognition), and treated as a reliable indicator of the participants’ memory representation of the visual scene.

Next, we computed a fourth score, namely, the percentage of confident responses irrespective of memory retrieval performance (‘Sure’), which represents a measure of the general tendency of the participants to make a “Sure” judgment irrespective of memory performance. Finally, to evaluate how good the participants were at judging the correctness of their responses, we computed a ‘Metacognitive sensitivity’ index (type 2 d-prime) according to the formula: type 2 d-prime = z (type 2 hits rate) − z (type 2 false alarms rate)^63,64^. “Type 2 hits rate” was defined as the proportion of trials in which participants reported “Sure” judgment within the total amount of correct responses; while “type 2 false alarms rate” was defined as the proportion of trials in which participants reported “Sure” judgment within the total amount of incorrect responses.

Five mixed-analyses of variance (ANOVAs) were used to evaluate whether each of the dependent variables (i.e., ‘Correct’, ‘Accuracy sensitivity’, ‘Correct & Sure’, ‘Sure’, and ‘Metacognitive sensitivity’) varied as a function of the between-participants factor of age group (3 levels: young children, older children, and adults), and the within-participants factor of target saliency (2 levels: Max and Min).

Moreover, we examined the possibility that the complexity of the visual scenes, as operationalized by the NSL index, would have a predictive effect on the performance at the visuo-spatial STM task. To this aim, for each scene, we computed the NSL (see the “Stimuli and task” section, above), and each of the previous dependent variables tested with the ANOVAs (i.e., ‘Correct’, ‘Accuracy sensitivity’, ‘Correct & Sure’, ‘Sure’, and ‘Metacognitive sensitivity’ indexes) separately for each of the three age groups. For these analyses, ‘Correct’ scores were operationalized as the percentage of participants that correctly performed the memory retrieval task for each scene, irrespective of the confidence judgment. The computation of the ‘Accuracy sensitivity’ for each scene followed the classical formula d-prime = z (hits rate) − z (false alarms rate), where the hits rate was the proportion of participants who correctly identified the target appearing in the same position as in the original scene, while the false alarms rate was the proportion of participants who incorrectly identified the target appearing in a different position as matching the original scene. ‘Correct & Sure’ scores were operationalized as the percentage of participants that provided a correct and sure response for each scene. ‘Sure’ scores were operationalized as the percentage of participants that reported to be sure for each scene, irrespective of memory retrieval performance. Finally, the computation of the ‘Metacognitive sensitivity’ for each scene followed the formula type 2 d-prime = z (type 2 hits rate) − z (type 2 false alarms rate), where type 2 hits rate was the proportion of participants who reported “Sure” judgment given their response was correct, while type 2 false alarms rate was the proportion of participants who reported “Sure” judgment given their response was incorrect. After having computed these data, we adopted a hierarchical multiple regressions approach. Five separate four-step hierarchical multiple regressions were conducted to identify whether the NSL, and the two-way interaction terms (i.e., “NSL by target saliency” and “NSL by age group”) explained a statistically significant amount of variance in each of the five dependent variables. Model 1 of each hierarchical regression was based on the related significant effects revealed by the ANOVAs described above (see also the Results section, below). Accordingly, Model 1 for the ‘Correct’, and the ‘Accuracy sensitivity’ scores included only the dummy variables representing the age groups (young children, older children, and adults) and the target saliency (Max and Min). On the contrary, Model 1 for ‘Correct & Sure’, ‘Sure’, and ‘Metacognitive sensitivity’ scores, included the dummy variables representing age groups, the dummy variables representing the target saliency, and also their related two-way interaction term. In all hierarchical regressions, the NSL was added in Model 2, as a continuous predictor; the interaction term between the NSL and the dummy variables representing the target saliency was added in Model 3; and, finally, the interaction term between the NSL and the dummy variables representing the age groups was added in Model 4. Before running these analyses, we checked that the number of observations, 144 (i.e., 48 scenes × 3 age groups), was enough to guarantee sufficient statistical power for the regression models. This was confirmed by a power analysis that indicated a minimum number of 111 observations, taking into account a conservative effect size of 0.20, a power of 95%, a significance level of 0.05, and 6 predictors (Age group, Saliency, NSL, and the related interaction terms).

All the statistical analyses were performed using RStudio 1.3.1073 (https://www.rstudio.com/). Reported p-values were corrected for multiple comparisons using the Holm-Bonferroni correction.

## Results

**Figure 2:**
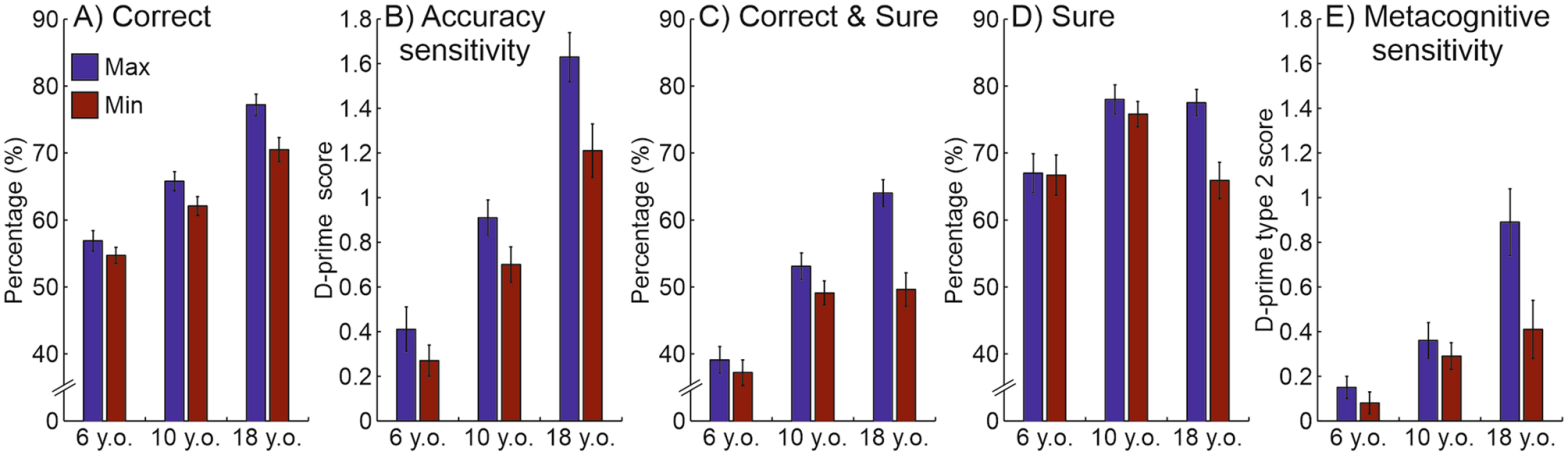
Bar graphs displaying mean performance as a function of age groups and saliency conditions. Mean values of: (**A**) correct responses irrespective of confidence, (**B**) accuracy sensitivity, (**C**) correct and sure responses, (**D**) sure responses irrespective of accuracy, (**E**) metacognitive sensitivity. In all bar graphs, the error bars represent the standard error of the mean.

The results related to the five measured variables (‘Correct’, ‘Accuracy sensitivity’, ‘Correct & Sure’, ‘Sure’, and ‘Metacognitive sensitivity’) are illustrated in Fig. 2. Preliminarily, to ensure that all groups performed better-than-chance at the STM task in each of the two saliency conditions, we run a series of right-side one-sample t-tests on the ‘Correct’ and ‘Accuracy sensitivity’ data. All the t-tests on the ‘Correct’ scores revealed better-than-chance performance, i.e., a score significantly higher than 50%, in each group and for each saliency condition (young children Max saliency: *t*(62) = 4.41, *p* < .001; young children Min saliency: *t*(62) = 3.73, *p* < .001; older children Max saliency: *t*(66) = 11.61, *p* < .001; older children Min saliency: *t*(66) = 8.72, *p* < .001; adults Max saliency: *t*(38) = 17.10, *p* < .001; adults Min saliency: *t*(38) = 11.20, *p* < .001). Consistently, the t-tests on the ‘Accuracy sensitivity’ scores confirmed better-than-chance performance, i.e., a score significantly higher than 0, in each group and for each saliency condition (young children Max saliency: *t*(62) = 4.21, *p* < .001; young children Min saliency: *t*(62) = 3.63, *p* < .001; older children Max saliency: *t*(66) = 11.25, *p* < .001; older children Min saliency: *t*(66) = 8.54, *p* < .001; adults Max saliency: *t*(38) = 14.99, *p* < .001; adults Min saliency: *t*(38) = 10.16, *p* < .001).

Having established that the task was doable for all age groups, we conducted the planned ANOVAs. The ANOVA on the ‘Correct’ responses (see Fig. 2A) revealed a main effect of age group [*F*(2, 166) = 49.08, *p* < .001, η^2^_p_ = 0.372]. Post-hoc comparisons showed lower accuracy for the young children (Mean ± SE, 54.4 ± 1.2%) as compared with the older children (62.6 ± 1.2%; *p* < .001), who, in turn, showed lower accuracy than adults (72.5 ± 1.3%; *p* < .001). The ANOVA also revealed a main effect of target saliency [*F*(1, 166) = 16.26, *p* < .001, η^2^_p_ = 0.089], indicating higher accuracy for the Max (65.3 ± 0.9%) than for the Min condition (61.1 ± 0.9%). This analysis did not reveal any interaction between age group and target saliency [*F*(2, 166) = 1.43, *p* = .241, η^2^_p_ = 0.017], thus suggesting a comparable effect of perceptual saliency on memory performance across the three groups studied. This pattern of results was confirmed by the ANOVA on the ‘Accuracy sensitivity’ scores (the d-prime, see Fig. 2B) that revealed both the main effect of age group [*F*(2, 166) = 46.09, *p* < .001, η^2^_p_ = 0.357] and target saliency [*F*(1, 166) = 16.44, *p* < .001, η^2^_p_ = 0.090], but not the interaction effect [*F*(2, 166) = 1.59, *p* = .208, η^2^_p_ = 0.019]. However, when analysing ‘Correct & Sure’ responses a different picture emerges (see Fig. 2C). On one hand, this ANOVA confirmed the two main effects: age group [*F*(2, 166) = 26.63, *p* < .001, η^2^_p_ = 0.243], indicating a lower percentage of ‘Correct & Sure’ responses in young children (37.1 ± 1.8%) compared with older children (50.0 ± 1.8%; *p* < .001), whose, in turn, scored lower than adults (55.7 ± 1.9%; *p* = .037); and target saliency [*F*(1, 166) = 35.87, *p* < .001, η^2^_p_ = 0.178], indicating higher accuracy for the Max (51.0 ± 1.2%) than for the Min condition (44.2 ± 1.2%). On the other hand, this analysis also revealed a significant interaction between the two factors, age group and target saliency [*F*(2, 166) = 10.05, *p* < .001, η^2^_p_ = 0.108]. Post-hoc comparisons revealed a selective benefit of saliency in the adult group. Thus, when considering a combined measure of correct and confident responses (‘Correct & Sure’), only adult participants demonstrated a significant STM benefit in terms of target-related saliency (Max vs. Min; 64.0 ± 2.1% vs. 49.6 ± 2.5%, *p* < .001), while young children and older children showed similar memory performance irrespective of Max versus Min target salience (39.1 ± 2.0% vs. 37.2 ± 1.9%, *p* = .805; and 53.1 ± 2.0% vs. 49.1 ± 1.8%, *p* = .100, for young and older children, respectively).

Then, we tested whether age group and target saliency had an impact on the percentage of ‘Sure’ responses (see Fig. 2D). This ANOVA revealed a main effect of target saliency [*F*(1, 166) = 26.75, *p* < .001, η^2^_p_ = 0.139], indicating a higher rate of ‘Sure’ responses in the Max (74.3 ± 1.5%) than in the Min condition (69.6 ± 1.5%). The ANOVA also revealed a main effect of age group [*F*(2, 166) = 4.88, *p* = .009, η^2^_p_ = 0.056]. Post-hoc comparisons indicated a reduced amount of ‘Sure’ responses in young (67.0 ± 2.4%) than in older children (77.0 ± 2.4%; *p* = .006). Surprisingly, no significant difference was detected between older children and adults (71.9 ± 2.6%; *p* = .330), but this was clarified by a significant age group by target saliency interaction [*F*(2, 166) = 12.84, *p* < .001, η^2^_p_ = 0.134], showing that the target saliency had a different impact on the confidence ratings depending on the participants’ age. Post-hoc comparisons revealed that the percentage of ‘Sure’ responses was affected by the target salience only in the adult group, with a higher rate of ‘Sure’ responses in the Max than in the Min condition (77.5 ± 2.0% vs. 65.9 ± 2.7%, *p* < .001), but not so in the other two age groups (both *p*s > .701).

Finally, we explored the participants’ ability to judge their own responses through a ‘Metacognitive sensitivity’ index (type 2 d-prime), and tested whether this metacognitive ability varied according to age groups and target salience conditions (see Fig. 2E). The ANOVA conducted on the ‘Metacognitive sensitivity’ data showed once again the main effect of age group [*F*(2, 165) = 15.81, *p* < .001, η^2^_p_ = 0.161] and target saliency [*F*(1, 165) = 10.97, *p* < .001, η^2^_p_ = 0.062], suggesting that the ability to proper evaluate one’s own memory performance changed across age and target saliency conditions. Post-hoc comparisons showed a higher ‘Metacognitive sensitivity’ in the young adults (0.60 ± 0.07) compared with the older children group (0.28 ± 0.06; *p* = .001) that, in turn, was higher than in the young children group (0.07 ± 0.06; *p* = .011). Moreover, ‘Metacognitive sensitivity’ was higher in the Max than in the Min target saliency condition (0.42 ± 0.05 and 0.21 ± 0.05; for Max and Min condition, respectively). This analysis also revealed a significant interaction between age group and target saliency [*F*(2, 165) = 3.99, *p* = .020, η^2^_p_ = 0.046]. Post-hoc comparisons revealed a selective benefit, driven by target salience, in the adult group when judging their own memory responses. Indeed, only adults demonstrated a significant enhancement of Metacognitive sensitivity for Max versus Min targets (0.89 ± 0.15% vs. 0.41 ± 0.13, *p* = .003), while young and older children showed comparable Metacognitive sensitivity irrespective of target salience (0.15 ± 0.05 vs. 0.07 ± 0.05, *p* = 1.000; and 0.35 ± 0.08 vs. 0.29 ± 0.06, *p* = 1.000, for young and older children, respectively).

**Figure 3:**
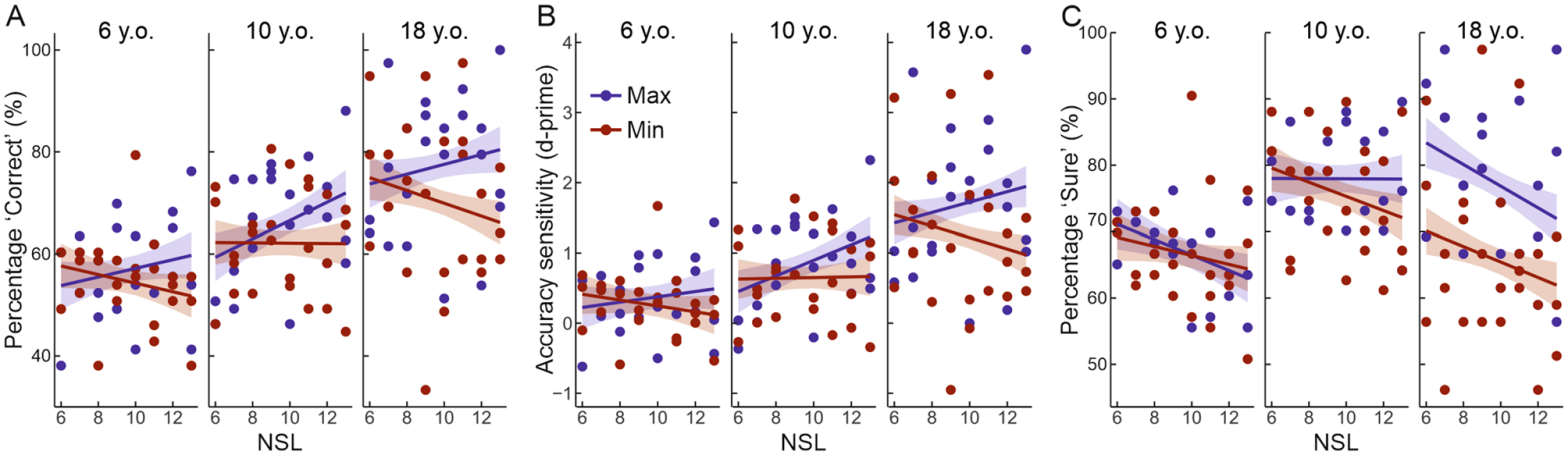
Scatterplots displaying the percentage of ‘Correct’ responses (panel **A**), the accuracy sensitivity (panel **B**), and the percentage of ‘Sure’ responses (panel **C**) as a function of the “number of salient locations” (NSL) in the different age groups and target salience conditions. In all graphs, the coloured bands around the regression lines represent the standard error of the mean.

Finally, we conducted hierarchical regressions to examine whether the ‘Correct’, ‘Accuracy sensitivity’, ‘Correct & Sure’, ‘Sure’, and ‘Metacognitive sensitivity’ scores in the different age groups and target saliency conditions could be predicted by scene complexity, i.e., the NSL index (see Tables 1, 2, 3, 4, and 5). Concerning the ‘Correct’ data (see Table 1), the addition of the NSL predictor (Model 2) did not significantly improve the predictive power of the model [*F*(1, 136) = 0.17, *p* = .680]. However, the addition of the interaction term between NSL and the dummy variables representing the target saliency (Model 3) significantly improved the predictive power of the model [*F*(1, 135) = 4.92, *p* = .028]. Model 3 showed a significant interaction between target saliency and the NSL index (β = − 0.702, *p* = .028), suggesting that the effect of target saliency on memory accuracy was affected by the complexity of the scenes (i.e., for the Max condition, the higher the NSL, the higher the accuracy; while for the Min condition, the higher the NSL, the lower the accuracy, see Fig. 3A). The addition of the interaction term between NSL and the dummy variables representing the age groups in Model 4 did not significantly improve the predictive power of the model [*F*(2, 133) = 0.56, *p* = .573]. These results were fully confirmed by the hierarchical regressions with the “Accuracy sensitivity” score (see Fig. 3B, and Table 2), wherein the addition of the interaction term between NSL and the target saliency (Model 3) significantly improved the predictive power of the model [*F*(1, 135) = 4.11, *p* = .045].

**Table 1:** Summary of hierarchical regression analyses for variables predicting ‘Correct’ responses.

	Model 1	Model 2	Model 3	Model 4
**Predictors**				
Age group				
Young children versus older children	0.280***	0.280***	0.280***	− 0.007
Young children versus adults	0.614***	0.614***	0.614***	0.666
Saliency				
Max versus min	− 0.152*	− 0.151*	0.513	0.513
NSL		0.029	0.197	0.158
Interaction term				
NSL × min saliency			− 0.702*	− 0.702*
NSL × older children group				0.299
NSL × adults group				− 0.054
**R**^**2**^	0.291	0.287	0.307	0.302
**ΔR**^**2**^		− 0.004	0.020	− 0.005
**F for change in R**^**2**^		0.17	4.92*	0.56

Standardized regression coefficients (β) are reported for each predictor.

The age group was presented as three dummy variables (young children, older children, and adults) with young children serving as the reference group.

The saliency condition was presented as two dummy variables (Max and Min) with Max saliency serving as reference condition.

*p < .05, **p < .01, ***p < .001.

**Table 2:** Summary of hierarchical regression analyses for variables predicting the ‘Accuracy sensitivity’ index.

	Model 1	Model 2	Model 3	Model 4
**Predictors**				
Age group				
Young children versus older children	0.230**	0.230**	0.230**	− 0.071
Young children versus adults	0.609***	0.609***	0.609***	0.629
Saliency				
Max versus min	− 0.138	− 0.137	0.473	0.473
NSL		0.034	0.189	0.142
Interaction term				
NSL × min saliency			− 0.645*	− 0.645*
NSL × older children group				0.314
NSL × adults group				− 0.020
**R**^**2**^	0.288	0.284	0.300	0.295
**ΔR**^**2**^		− 0.004	0.016	− 0.005
**F for change in R**^**2**^		0.23	4.11*	0.54

Standardized regression coefficients (β) are reported for each predictor.

The age group was presented as three dummy variables (young children, older children, and adults) with young children serving as the reference group.

The saliency condition was presented as two dummy variables (Max and Min) with Max saliency serving as reference condition.

*p < .05, **p < .01, ***p < .001.

**Table 3:** Summary of hierarchical regression analyses for variables predicting ‘Correct & Sure’ responses.

	Model 1	Model 2	Model 3	Model 4
**Predictors**				
Age group				
Young children versus older children	0.398**	0.398**	0.398**	0.182
Young children versus adults	0.710***	0.710***	0.710***	0.859*
Saliency				
Max versus min	− 0.057	− 0.058	0.312	0.312
NSL		− 0.048	0.045	0.034
Interaction term				
Older children group × min saliency	− 0.047	− 0.047	− 0.047	− 0.046
Adults group × min saliency	− 0.288*	− 0.288*	− 0.288*	− 0.288*
NSL × min saliency			− 0.391	− 0.391
NSL × older children group				0.224
NSL × adults group				− 0.155
**R**^**2**^	0.257	0.254	0.256	0.251
**ΔR**^**2**^		− 0.003	0.002	− 0.005
**F for change in R**^**2**^		0.43	1.42	0.52

Standardized regression coefficients (β) are reported for each predictor.

The age group was presented as three dummy variables (young children, older children, and adults) with young children serving as the reference group.

The saliency condition was presented as two dummy variables (Max and Min) with Max saliency serving as reference condition.

*p < .05, **p < .01, ***p < .001.

**Table 4:** Summary of the hierarchical regression analyses for variables predicting ‘Sure’ responses.

	Model 1	Model 2	Model 3	Model 4
**Predictors**				
Age group				
Young children versus older children	0.483***	0.483***	0.483***	0.346
Young children versus adults	0.463***	0.463***	0.463***	0.666
Saliency				
Max versus min	− 0.010	− 0.012	0.003	0.003
NSL		− 0.200**	− 0.197	− 0.186
Interaction term				
Older children group × min saliency	− 0.070	− 0.070	− 0.070	− 0.070
Adults group × min saliency	− 0.403**	− 0.403**	− 0.403**	− 0.404**
NSL × min saliency			− 0.015	− 0.015
NSL × older children group				0.142
NSL × adults group				− 0.210
**R**^**2**^	0.215	0.251	0.246	0.239
**ΔR**^**2**^		0.036	− 0.005	− 0.007
**F for change in R**^**2**^		7.39**	0.00	0.44

Standardized regression coefficients (β) are reported for each predictor.

The age group was presented as three dummy variables (young children, older children, and adults) with young children serving as reference group.

The saliency condition was presented as two dummy variables (Max and Min) with Max saliency serving as reference condition.

*p < .05, **p < .01, ***p < .001.

**Table 5:** Summary of the hierarchical regression analyses for variables predicting the ‘Metacognitive sensitivity’ index.

	Model 1	Model 2	Model 3	Model 4
**Predictors**				
Age group				
Young children versus older children	0.122	0.122	0.122	0.064
Young children versus adults	0.524***	0.524***	0.524***	0.696
Saliency				
Max versus min	− 0.072	− 0.072	− 0.086	− 0.091
NSL		0.005	0.001	0.018
Interaction term				
Older children group × min saliency	0.032	0.032	0.032	− 0.033
Adults group × min saliency	− 0.169	− 0.169	− 0.169	− 0.168
NSL × min saliency			0.015	0.020
NSL × older children group				0.061
NSL × adults group				− 0.180
**R**^**2**^	0.129	0.122	0.116	0.105
**ΔR**^**2**^		− 0.007	− 0.006	− 0.011
**F for change in R**^**2**^		0.00	0.00	0.19

Standardized regression coefficients (β) are reported for each predictor.

The age group was presented as three dummy variables (young children, older children, and adults) with young children serving as reference group.

The saliency condition was presented as two dummy variables (Max and Min) with Max saliency serving as reference condition.

*p < .05, **p < .01, ***p < .001.

Concerning the ‘Sure’ data (see Table 4), the addition of NSL (Model 2) significantly improved the predictive power of the model [*F*(1, 134) = 7.39, *p* = .007], revealing a decrease in the confidence rating as a function of scene complexity (β = − 0.200, *p* = .007), i.e., the higher the NSL, the lower the rate of confident responses (see Fig. 3C). The addition of the interaction term between NSL and the dummy variables representing the target saliency in Model 3, and the addition of the interaction term between the NSL and the dummy variables representing the age groups in Model 4 did not significantly improve the model fitting ([*F*(1, 133) = 0.00, *p* = .963], and [*F*(2, 131) = 0.44, *p* = .642]; respectively). Finally, the analyses on the ‘Correct & Sure’ and ‘Metacognitive sensitivity’ data did not appear to be affected by the scene complexity, since no one of the additions improved the predictive power of Model 1 (‘Correct and Sure’: all *p*s > .236, see Table 3; ‘Metacognitive sensitivity’: all *p*s > .830, see Table 5).

## Discussion

The main aim of the current study was to investigate whether perceptual salience affects STM performance and meta-memory skills to a similar or a different extent from childhood to adulthood. STM performance and meta-memory abilities of 6, 10, and 18 y.o. participants were assessed using a delayed match-to-sample task, in which we manipulated—at encoding—the perceptual salience (maximal vs. minimal) of the target object to be discriminated at retrieval. We computed several indices of performance, related to the participants’ accuracy at the spatial discrimination task performed at retrieval and to the participants’ metacognitive (confidence) judgments. Overall, the analyses showed that both memory performance and meta-memory skills are strongly affected by the participants’ age and target saliency.

Consistently with previous literature, we found that STM performance improved as a function of age (see, e.g.,^23–25,27,65,66^; for a review, see^28^). Our data showed a linear increase in performance moving from 6, to 10 and 18 y.o. participants, both in terms of ‘Correct’ and ‘Correct & Sure’ responses, as well as in terms of ‘Accuracy sensitivity’ (cf. Fig. 2A–C). Analogously, regarding metacognition, we observed that the age level significantly modulated both meta-memory judgments (i.e., the response confidence) and the correct estimation of such judgments (i.e., the ‘Metacognitive sensitivity’ score). The raw percentage of ‘Sure’ responses significantly increased from young to older children (cf. Fig. 2D), and, surprisingly, no differences were found between older children and adults in the percentage of ‘Sure’ responses. However, qualitatively different mechanisms emerge from the two older age groups (10 and 18 y.o. participants), when considering the ability to correctly judge own memory performance as measured through the ‘Metacognitive sensitivity’ score. In fact, adults showed a greater capability to correctly judge their own memory performance than 10 y.o. children, who in turn outperformed younger 6 y.o. children (cf. Fig. 2E). This latter finding highlights clear developmental differences between children and young adults not only in terms of STM performance, but also in relation to metacognitive capabilities to properly judge the content of one’s own memory representation. Metacognitive skills are fundamental to promote the individual’s cognitive development, both in general (e.g.^48,49,55^) and specifically in relation to STM/working memory development^53,54^. For instance, Forsberg and colleagues^54^ compared meta-working memory judgments to actual performance in children between 6 and 13 y.o. and adults. They found an overestimation of meta-memory judgments accuracy in younger participants concerning their memory performance, suggesting a later development of memory-related metacognitive skills. Consistently with this latter finding, our results revealed a lower capacity in children of 6 and 10 y.o. to correctly estimate their memory performance compared to adults and showed that at the developmental stages taken into account (6, 10, and 18 years) correspond a progressive increase in the ability to correctly judge one’s own memory accuracy.

Our design also allowed us to assess the impact of encoding-related perceptual salience on STM retrieval and meta-memory skills. When considering ‘pure’ accuracy and accuracy sensitivity, STM performance increased for targets at maximal versus minimal salience irrespective of the age group (cf. Fig. 2A, B). This finding indicates that perceptual salience affected to an equal extent STM performance at the different developmental stages taken into account. Since salient objects were better recognized by each age group (cf. the main effect of target saliency), we might reasonably assume that the maximal salient objects were more easily encoded (prioritized and processed) over the minimal salient objects in each age group. Crucially, however, when considering a more refined measure of memory performance that takes into account, combining together, both memory performance and meta-memory judgments (i.e., excluding all correct but ‘unsure’ trials, in which the participants had likely no memory of the target position but they were anyway forced to provide a response), the picture remarkably changed. Specifically, the analysis of ‘Correct & Sure’ responses revealed that only adults showed enhanced STM performance when—at retrieval—the spatial discrimination task involved a target that—at encoding—was located on the maximal salience location of the scene as compared to the location of minimal salience (cf. Fig. 2C). The saliency effect on STM performance in adults is in agreement with the previous literature, wherein a consistent STM advantage was found for the retrieval of higher versus lower salience targets (^14–20^; see, for a review^13^). On the other hand, the current results revealed no impact of perceptual salience on STM performance at earlier and later childhood (6 and 10 years, respectively). This latter finding appears to be consistent with those studies supporting an increased influence of perceptual salience to predict eye movements and overt exploration as a function of age^29,31–35^. However, it might be also possible that—as an alternative account—performance differences between children and adults arose not at the encoding, but at the following stages, namely, at maintenance, e.g. as a consequence of a different memory temporal decay, or at retrieval, e.g. due to a different precision of memory reactivation and representation (see, e.g.^27,67,68^).

Our findings also revealed an interplay between age and target saliency concerning meta-memory judgments and metacognitive sensitivity. In fact, only in the adult group, the percentage of ‘Sure’ responses increased—at retrieval—when the to-be-judged target was presented—at encoding—in a maximal versus minimal salient location of the scene (cf. Fig. 2D). This finding indicates a late development in the link between items that are easier to be processed/encoded and the related confident judgment (i.e., the encoding fluency effect^45,46^). Analogously, only in the adult group, ‘Metacognitive sensitivity’ was higher when the judgment pertains to the targets that—at encoding—were located in a maximal versus minimal salient location, while the correctness of metacognitive judgment in young and old children was not affected by the saliency of the target (cf. Fig. 2E). Overall, these findings further extend the notion that the accuracy of meta-memory judgments lately develops across age^54^, and indicate that additional factors, such as perceptual salience, can significantly contribute to the development of metacognitive skills related to the judgment of the content of one’s own memory representation.

Finally, we tested whether the access to STM was affected by scene complexity, as indexed by the number of salient locations (NSL) of the scene (for a similar approach, see^56^). In contrast with our original predictions, we did not find decreased STM performance when scene complexity increases. Conversely, we found that the NSL affected the percentage of ‘Correct’ responses and the ‘Accuracy sensitivity’ differently in the saliency conditions (cf. Fig. 3A, B). The higher the NSL the higher the participants’ memory performance at the STM task (i.e., a higher percentage of correct responses and higher accuracy sensitivity) for targets at maximal salience. By contrast, the higher the NSL the lower the participant’s memory performance at the STM for targets at minimal salience. This suggests that the benefit of perceptual salience increases as a function of the complexity of the visual scene: the more the scene is complex and the more the selection and the encoding of high salience objects is prioritized over the other, lower salience objects (e.g.^1–3^). The NSL was also found to predict a decrease of ‘Sure’ responses, indicating that the higher the complexity of the scene, the lower the confidence in judging own memory performance (cf. Fig. 3C). This finding is in line with the observed “dispersion” of attentional resources when coping with highly complex stimuli^56^, which appears to affect subsequent memory-related confidence judgments. Taken together, these latter findings highlight general constraints of complex visual scenes to the selection, encoding, and retrieval performance irrespective of the participants’ age.

It might be worth noting some limitations of the current study. Here we focused on two specific age groups of 6 and 10 y.o. children, while taking into account a larger spectrum of ages would allow gleaning a more complete picture of these processes. Another important limitation relies on the lack of acquisition of participants’ eye movements, which would allow assessing more precisely the impact of perceptual salience during the encoding phase of the visual scenes in each age group.

To conclude, we provided initial evidence of developmental differences related to the impact of perceptual salience on STM performance and meta-memory skills from early to later childhood and young adulthood, with significant salience-related effects only in adult participants.

## Data Availability

The dataset generated and analysed during the current study is available from the corresponding author on reasonable request.
